# LncRNAs expression signatures of human brain arteriovenous malformation revealed by microarray

**DOI:** 10.1097/MD.0000000000011308

**Published:** 2018-07-27

**Authors:** Xiong Li, FuXin Lin, Jun Wu, Shuo Wang

**Affiliations:** a Department of Neurosurgery, Beijing ChaoYang Hospital; b Department of Neurosurgery, Beijing Tiantan Hospital, Capital Medical University.

**Keywords:** AVM, expression profiling analysis, lncRNA

## Abstract

Long noncoding RNAs (LncRNAs) were important genes involved in a variety of biological functions. They are aberrantly expressed in many types of diseases. In this study, we described LncRNAs profiles in 4 pairs of human brain arteriovenous malformation(AVM) and the corresponding fragment of superior temporal arteries(STA) or small scalp arteries (controlled arteries, CA) and try to find LncRNAs that correlated with the human brain AVM and with clinical symptoms.

4 pairs of AVM tissues and corresponding STA or scalp artery fragments (depended on the operative approach) of 4 AVM patients who were admitted in Beijing TianTan hospital were collected. Then LncRNA and mRNA expression profiling analysis was performed by Arraystar-LncRNA array. From the data, we found 1931 LncRNAs upregulated (>2 folds) and 1852 downregulated (<2 folds) in total 28,012 LncRNAs that could be detected. We also found 1577 upregulated mRNAs (>2 folds) and 1699 downregulated (<2 folds) in 21,780 mRNAs that could be detected. LncRNAs (ENST00000423394, ENST00000444114, TCONS_00013855, and ENST00000452148) were evaluated by qPCR in 14 pairs of AVM nidus and the control. This 4 LncRNAs were aberrantly expressed in AVM nidus compared with the control. LncRNA (ENST00000423394) correlated with epilepsy (*R* = 0.34, *P = *.02, 95% confidence interval 0.08–0.85)

We found that development of AVM may correspond with downregulation of NADPH reductase, lipoprotein lipase and Optic atrophy related proteins. It also may correspond with upregulation of Fcγreceptor. The downregulation of NADPH reductase may correlate with seizures of AVM patients.

## 1 Introduction

Cerebral arteriovenous malformations were vascular lesions characterized by direct blood shunting from artery to the vein without capillary bed.^[1]^ The causation and development of AVM were not clear.^[2–5]^ Previous research showed that the process of cerebral arteriovenous malformation involved a large variety of genetic, mocular, and biological factors. LncRNAs were defined as noncoding RNAs that longer than 200 nucleotides in length and had been validated to have comprehensive function in biological processes through several of mechanism.^[6,7]^ Misregulation of LncRNAs has been showed to be associated with many human diseases.^[8,9]^ There was rare study on AVM by using LncRNA microarray technique. Here we report our exploration of the LncRNAs of brain AVM.

## 2 Material and method

### 2.1 Sample collection

All the patients were admitted in Beijing Tiantan Hospital from April, 2013 to January, 2015 and had signed the statement of consenting to collect samples from them. There were 9 males and 5 females of these patients, from 11 years old to 45 years old (average 27.3 years). All the patients had been certified as cerebral arteriovenous malformation by digital subtraction angiography. Brain AVM tissues from14 patients were collected and 14 STA or small scalp arteries fragments from same patients were also collected as control. The tissues and the control fragments were put in the liquid nitrogen immediately after resection. This study had been approved by the ethics committee of Beijing Tiantan Hospital and had been conducted according to the principles expressed in the Declaration of Helsinki. First, we perform expression analysis on 4 pair samples. Then we chose 8 LncRNAs that expressed most differently in profiling analysis and then verified them by qPCR in total 14 pairs samples. RNA quantity and quality were measured by NanoDrop ND-1000. RNA integrity was assessed by standard denaturing agarose gel electrophoresis.

### 2.2 Preparations for RNA and cDNA

Tissues were collected in phosphate buffered saline (PBS; 0.9% NaCl in 0.01 M sodium phosphate buffer, pH 7.4), which had been treated with 0.1% diethyl pyrocarbonate (DEPC-PBS). After the wall of each piece of tissue had been opened, the tissue was cleaned with DEPC-PBS and transferred to Tirol (Invitrogen) for extraction of total RNA, which was isolated according to the manufacturer's instructions and stored at −80°C for later use. Samples of cDNA were generated by reverse transcription with 5 μg total RNA, 50 ng random hexamerprimers, 10 nM dNTPs, incubated at 65°C for 5 minutes, and placed on ice for at least 1 minutes, with the addition of 40 URNase OUT, 200U Superscript III RT, 10 mM dithiothreitole, and 5 mM MgCl_2_ (Invitrogen), in a 20 μL reaction volume. Following brief centrifugation, the reactions were incubated at 50°C for 50 minutes and then at 70°C for 15 men. The completed reverse transcription reactions were stored at −20°C and used for the polymerase chain reaction (PCR) without further treatment.

### 2.3 RNA labeling and array hybridization

Sample labeling and array hybridization were performed according to the Agilent One-Color Microarray-Based Gene Expression Analysis protocol (Agilent Technology) with minor modifications. Briefly, mRNA was purified from total RNA after removal of rRNA (mRNA-ONLY Eukaryotic mRNA Isolation Kit, Epicenter). Then, each sample was amplified and transcribed into fluorescent cRNA along the entire length of the transcripts without 3′ bias utilizing a random priming method. The labeled cRNAs were purified by RNeasy Mini Kit (Qiagen). The concentration and specific activity of the labeled cRNAs (pmol Cy3/μg cRNA) were measured by NanoDrop ND-1000. 1 μg of each labeled cRNA was fragmented by adding 5 μL 10 × Blocking Agent and 1 μL of 25 × Fragmentation Buffer, then heated the mixture at 60°C for 30 minutes, finally 25 μL 2 × GE Hybridization buffer was added to dilute the labeled cRNA. 50 μL of hybridization solution was dispensed into the gasket slide and assembled to the LncRNA expression microarray slide. The slides were incubated for 17 hours at 65°C in hybridization oven. The hybridized arrays were washed, fixed and scanned with using the Agilent DNA Microarray Scanner (part number G2505C).

### 2.4 Data analysis

Agilent Feature Extraction software (version 11.0.1.1) was used to analyze acquired array images. Quantile normalization and subsequent data processing were performed with using the GeneSpring GX v11.5.1 software package (Agilent Technologies). After quantile normalization of the raw data, LncRNAs and mRNAs that at least 4 out of 8 samples have flags in Present or Marginal (“All Targets Value”) were chosen for further data analysis. Differentially expressed LncRNAs and mRNAs with statistical significance between the 2 groups were identified through Volcano Plot filtering and differentially expressed LncRNAs/mRNAs were identified through Fold Change filtering. Hierarchical Clustering was performed using the Agilent GeneSpring GX software (version 11.5.1). GO analysis and pathway analysis were performed in the standard enrichment computation method.

### 2.5 Real-time PCR and sequencing

Based on the result of differential expression analysis of mRNA and GO analysis, we choose 8 LncRNAs for R-T PCR. Total RNA of the samples were extracted using the Trizol Reagent (Invitrogen, Carlsbad, CA) according to the protocol of the manufacturer. Primers for each gene had been listed in Table 1.

**Table 1:**
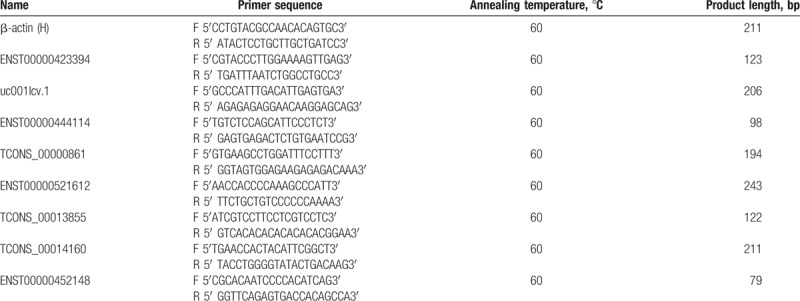
Primer for RT-PCR.

## 3 Result

### 3.1 Results were divided to 4 aspects

#### 3.1.1 Differentially expressed LncRNAs and mRNAs screening

**Figure 1:**
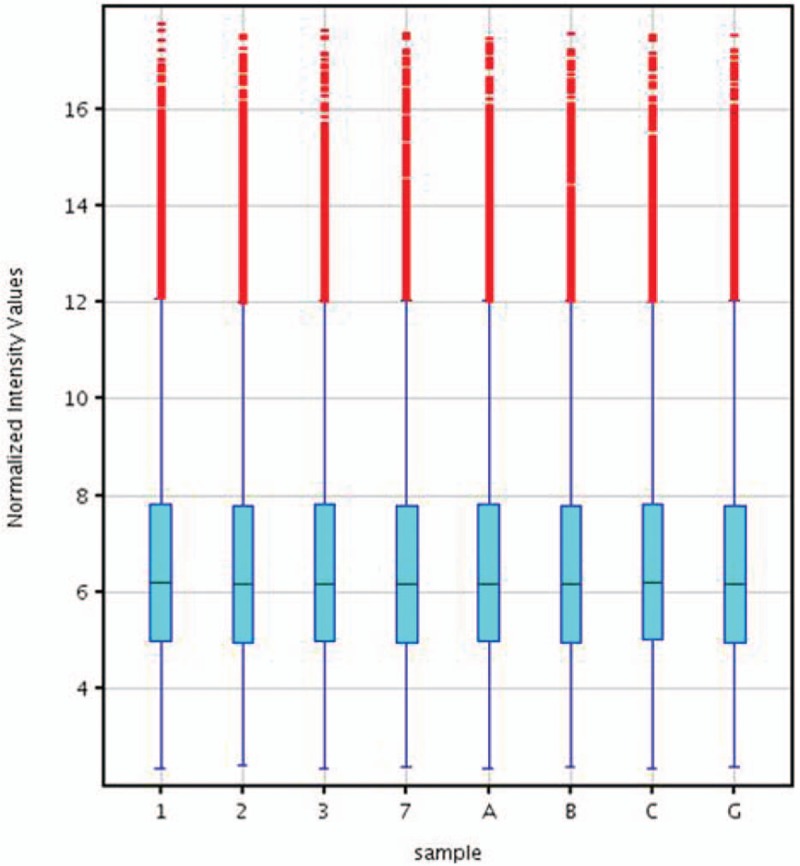
The box plot of samples legend: Box plot view is used to look at, and compare. The distributions of expression values for the samples in an experiment after normalization.

Quantile normalization and subsequent data processing were using the GeneSpring GX v11.5.1 software package (Agilent Technologies). After quantile normalization of the raw data, mRNAs that at least 4 out of 8 samples have flags in Present or Marginal (“All Targets Value—mRNAs”) were chosen for differentially expressed mRNAs screening (see Table 2). After filtering, we used Box-Plot to visualize the distributions of a dataset (Fig. 1). To identify significant differentially expressed mRNAs with statistical significance, we performed a Volcano Plot filtering between the 2 compared groups. The threshold is Fold Change > = 2.0 and *P*-value< = .05. The *P*-values calculated from paired *t*-test. From the data, we found 1931 LncRNAs unregulated (>2 folds), 450 LncRNAs (>4 folds) and 1852 LncRNAs (<2 folds), 719 LncRNAs (<4 folds) down regulated in 28012 LncRNAs that could be detected. We also found 1577 mRNAs (>2 folds), 450 mRNAs (>4 folds) upregulated, and 1699 (<2 folds), 681 mRNAs (<4 folds) downregulated in 21,780 mRNAs that could be detected (Table 3).

**Table 2:**
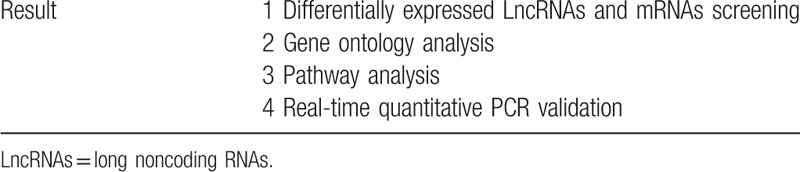
Briefing of experimental method.

**Table 3:**
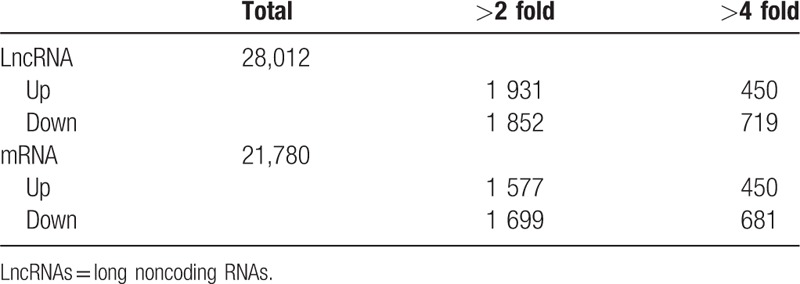
Numbers of significant differentially expressed LncRNAs and mRNAs.

#### 3.1.2 Gene ontology analysis

GO analysis was a functional analysis associating differentially expressed mRNAs with GO categories (see Table 4). The GO categories were derived from Gene Ontology (http://www.geneontology.org), which comprised 3 structured networks of defined terms that described gene product attributes. The ontology covered 3 domains: Biological Process, Cellular Component and Molecular Function. The *P*-value denoted the significance of GO Term enrichment in the differentially expressed mRNA list. *P*-value was calculated by Fisher's exact test (cut-off is .05) the less the *P*-value was, the more significant the GO Term was (*P*-value ≤.05 is recommended).

**Table 4:**
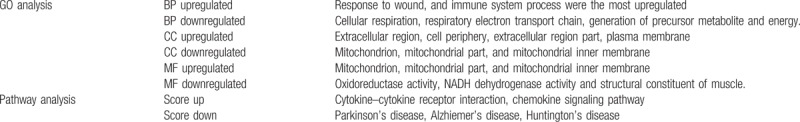
Briefing of Gene ontology analysis.

**Figure 2:**
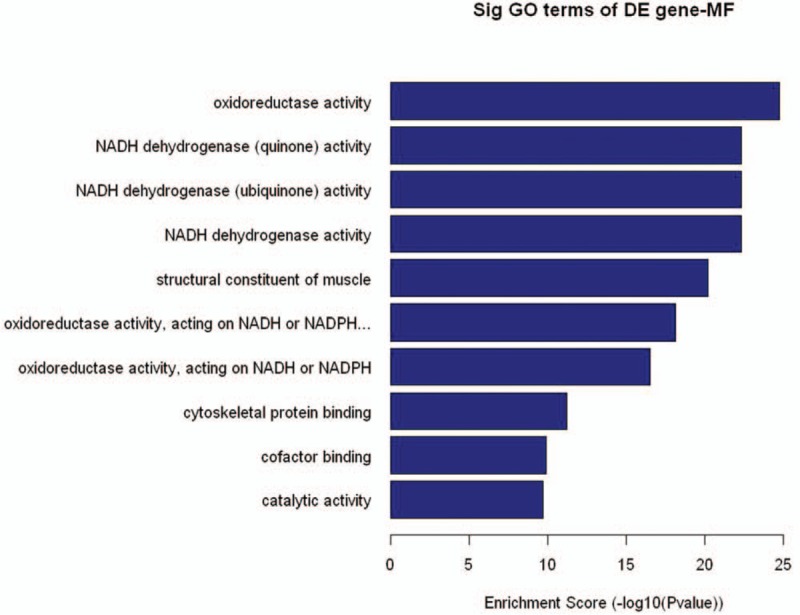
GO: gene ontology, MF: molecular function, DE: different expression, Enrichment. Score: The GOID‘s Enrichment Score value, it equals (−log10[*P*-value]).

During the up and downregulated terms of the 3 domains, we focus on the most downregulated terms in the molecular function analysis were: oxidoreductase activity, NADH dehydrogenase activity and structural constituent of muscle (Fig. 2).

#### 3.1.3 Pathway analysis

**Figure 3:**
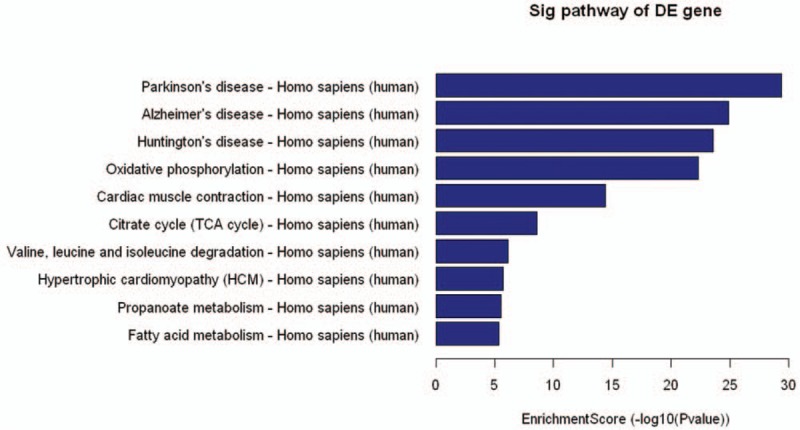
Enrichment Score: the Enrichment Score value of the Pathway, it equals (−log10[*P*-value]), DE: different expression.

Pathway analysis was a functional analysis mapping genes to KEGG pathways. The *P*-value (EASE-score, Fisher value or hyper geometric value) denoted the significance of the Pathway correlated to the conditions. Lower the *P*-value, more significant was the Pathway. (The recommend *P*-value cut-off is .05.). We could see mRNAs which downregulated in the pathway analysis (Fig. 3).

#### 3.1.4 Construction of the coding-noncoding gene co-expression network

LncRNAs and mRNAs with Pearson correlation coefficients not less than 0.99 were selected to draw the network by the program of cytoscape. We choose 8 mRNAs to be analyzed with the differential expressed LncRNAs. Among this CNC network, 476 pair nodes of co-expression LncRNAs and mRNAs composed the CNC network nodes. Most of pairs presented as positive correlation.

#### 3.1.5 Real-time quantitative PCR validation

Base on the CNC network, we choose 8 LncRNAs that most correlated with corresponding mRNAs. We found the qPCR data of lncRNA (ENST00000423394, *P = *.03), (ENST00000444114, *P = *.01), TCONS_00013855 (*P = *.01) and ENST00000452148 (*P = *.01) had strong consistence with the microarray data.

#### 3.1.6 Correlation analysis of (ENST00000423394) and clinical symptoms

From the CNC network analysis we found that each mRNAs correlated with several LncRNAs, but there was only one lncRNA (ENST00000423394) correlated with mRNA (FDXR). Pearson correlation analysis showed that lncRNA (ENST00000423394) had no correlation with clinical character of haemorrhage (*R* = 0.069, *P = *.36, 95% confidence interval 0.31–0.69), but correlated with epilepsy (R = 0.34, *P = *.02, 95% confidence interval 0.08–0.85)

## 4 Discussion

Brain arteriovenous malformation was characterized by high speed feeding artery, abnormal nidus and draining venous. Recent study shows that the development of AVM may be correlated with abnormal expression of gene, hemodynamic and angiogenesis factors. The development of AVM remained unclear due to complicated structure and rare occurrence. In this study, we try to find relationship between LncRNAs and AVMs.

In the previous study, researchers prefer to choose cells as control group derived from normal cortex of patients who underwent surgery for epilepsy. We choose the vessel tissues derived same patients as control for more strict and scientific contrast.

Previous gene research of brain arterivenous malformation showed that the development of AVM correlated with protein combination, receptor activation, actin binding, changing of activity of transferase and vascular endothelial growth.^[10]^ Other researchers showed that the path ways of cellular adhesion molecules, tight connection, cellular skeleton and MAPK may play important role development.^[11,12]^ Shenkar et al^[13]^ compared genes expression in cerebral cavernous malformation(CCMs) and AVMs to control superficial temporal arteries. They found 11 genes significantly differential expressed that related with angiogenesis factors, receptors and structure proteins. Takaqi et al^[14]^ categorized the genes with altered expression of human brain AVMs into 4 groups: death-related, neuron-related, inflammation-related, and other. Recently, Ferreira et al^[15]^ tried to use microRNA-18a as a therapeutic agent to improve AVM-BEC function. Compared to the researches listed before, we also found that the upregulation of Fcγ correlated with AVM which verified by the PCR. On the other way, we also found the development of AVM may correlate with the downregulation of LncRNAs that associated with NADPH, LPL, and OPA1.

**Figure 4:**
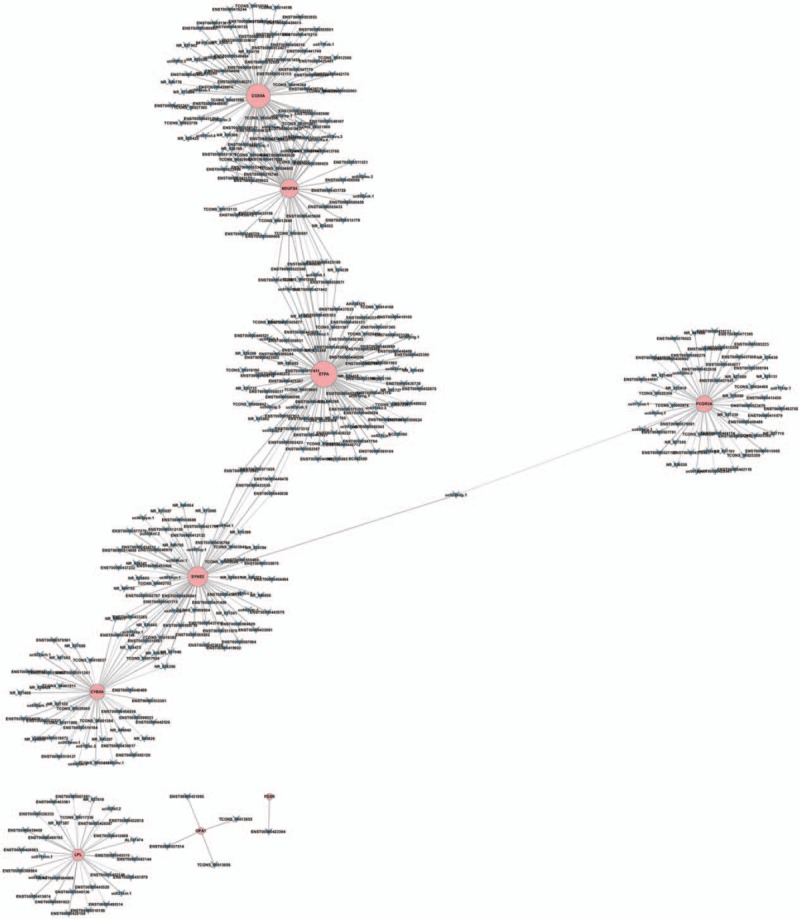
The pink node showed the mRNA, the blue V shape node represent the LncRNA. The active lines between the pink nodes and blue nodes mean positive regulation and dotted lines mean negative regulation between them.

The CNC network indicated that one mRNA could correlate with one to tens of LncRNAs and LncRNAs could correlate to 1 or 2 mRNAs (Fig. 4). Interestingly, there was only one lncRNA (ENST00000423394) correlated with mRNA (FDXR). The mRNA (FDXR) was known that its product was NADPH.NADPH participated in most oxidoreduction of carbohydrate, fat and protein and played important role in intracellular antioxidation. The reducing of NAD(P)H or phosphocreatine would inhibit the growth and differentiation of the cell and induce the apoptosis.^[16]^ Our gene ontology research showed the downregulation of LncRNA (ENST00000423394). The LncRNA (ENST00000423394) had positive relationship with mRNA (FDXR), who adjust the expression of NAD(P)H in the cells, may correlated with the development of AVM. The result was confirmed by the PCR test. NADPH was also marked as key role in delaying axonal degeneration which was known as main mechanism of neurodegenerative disease such as Parkinson disease (PD), Alzheimer disease, (AD), and Huntington disease (HD).^[17]^ The pathway analysis showed corresponding result that the development of AVM may correlate with the pathway of PD, AD and HD.

Our previous research found that the plasma level of IL-6 may be a predictor of hemorrhage of cerebral arteriovenous malformation. Zabel et al found that low blood mRNA level of TLR4 (toll-like receptor 4) and STAT3 (signal transducer and activator transcription 3) correlated with obliteration of AVM. Compare to these research, we found NADPH associated LncRNA (ENST00000423394) correlated with epilepsy but not hemorrhage in this research. Epilepsy was thought to result in generation of reactivity oxygen species (ROS) and inhibition of NADPH could reduce seizer-like activity-induced neuronal apoptosis. This supports the outcomes of our research.

OPA1 was a kind of mitochondria fusion related proteins which regulating the anaphase fusion of cells. Low expression of OPA1 may induce more splitting of mitochondria and raising the cell's sensibility to apoptosis. OPA1 may protect cells from apoptosis by inhibiting the release of cytochrom C.^[18]^ It also correlated with the stability of DNA and its location on the inner membrane of mitochondria was main place of oxidative phosphorylation. The mutation of OPA1 may reduce the energy metabolism. The OPA1RNAi cells showed obvious reduction of inner respiration and oxidative phosphorylation. This would lead to low output of ATP.^[19]^ Our research show the OPA1 associated LncRNA (TCONS_00013855) was downregulated which may reduce the stability of smooth muscle cells and pericyte of the vessels of AVM.

Take it together, after comparing 14 pairs LncRNAs and mRNAs of the AVM tissues and scalp artery fragment, we found that the development of AVM may correlated with the downregulation of OPA1 associated LncRNA(TCONS_00013855) and NADPH associated LncRNA (ENST00000423394). All these suggested the developing of AVM may not only correlate with the dysfunction structure but also with the deficiency of energy supply. There were 3 patients with epilepsy and we perform correlation analysis between the symptoms and the former LncRNAs. Interestingly, by Pearson correlation analysis we found LncRNA (ENST00000423394) correlated with clinical manifestation of epilepsy of the patients. There were 4 patients with hemorrhage, but we had not found correlation between this symptom with LncRNA (TCONS_00013855) and LncRNA (TCONS_00013855) and these afford new research orientation for us. Due to small sample size, there may be bias and we may lose important LncRNA during the selecting target RNAs from numerous choices.

## Author contributions

**Data curation:** xiong li.

**Investigation:** xiong li.

**Methodology:** xiong li.

**Resources:** fuxin lin, jun wu.

**Software:** xiong li.

**Supervision:** shuo wang.

**Writing – original draft:** xiong li.

**Writing – review & editing:** shuo wang.
